# A comparative evaluation of micro shear bond strength and microleakage between the resin-modified glass ionomer cement and residual dentin following excavation of carious dentin using Carie CareTM and conventional caries removal in primary teeth: an in vitro study

**DOI:** 10.12688/f1000research.131919.1

**Published:** 2023-03-24

**Authors:** Megha Nair, Arathi Rao, Jayaprakash Kukkila, Srikant Natarajan, Suprabha Baranya Srikrishna

**Affiliations:** 1 Pediatric and Preventive Dentistry, Manipal College of Dental Sciences, Mangalore, MAHE Manipal, Karnataka, 575001, India; 2 Department of Dental Materials, Biomaterials and Research Center, b. Yenepoya Dental College, Mangalore, Karnataka, 575018, India; 3 Oral Pathology, Manipal College of Dental Sciences, Mangalore, MAHE Manipal, Karnataka, 575001, India

**Keywords:** Carie CareTM, Primary teeth, residual dentin, resin modified glass iononmer

## Abstract

**Background:** The bond between the dentin and restorative material contributes to the success of the restoration. Structural changes associated with prepared dentin may influence the bonding of restorative materials. The present study evaluates the bond between the resin-modified glass ionomer cement (RMGIC) and residual dentin following excavation of carious dentin using Carie Care ^TM^ and conventional caries removal in primary teeth.

Methods:

52 primary teeth with dentinal caries were randomly grouped into group I, where caries removal was done using the conventional method, and group II which used Carie Care ^TM^. All the teeth were restored using RMGIC. Micro shear bond strength between the residual dentin and the cement was tested using universal testing machine and the dye penetration method was used for microleakage testing. Independent t-test was performed for intergroup comparison. Pearson chi-square test was carried out to evaluate the microleakage patterns in the enamel and dentin.

Results:

The mean micro-shear bond strength of group I was 6.03±1.6 and that of group II was 8.54±2.92; this difference was statistically significant with a *p*-value of 0.012. Microleakage was higher in the test group (1.38±0.51) than the control group (0.77±0.6) and was significant with a p *-*value of .036.

Conclusions:

Papain-based chemomechanical agent Carie Care ^TM^ can be used as an alternative method to conventional caries removal. However, further studies need to explore methods to improve the marginal sealing capacity of RMGIC to the residual dentin after chemomechanical caries removal.

## Introduction

Conventional caries removal method using rotary burs is easy and quick but has also been associated with unnecessary removal of affected dentin that could have been remineralized, patient discomfort and pain that may necessitate an administration of local anaesthesia. ^1^

To overcome these shortcomings, the chemomechanical caries removal (CMCR) system was introduced, forming the foundation of minimally invasive caries removal techniques. Carie Care ^TM^ is one such formulation containing purified papain enzyme. It was introduced in India by Vittal Mallya Scientific Research Foundation, Bengaluru, Karnataka, India, and Uni-Biotech Pharmaceuticals Pvt. Ltd in 2011. ^2^^,^^3^

The bond between the dentin and restorative material contributes to the success of the restoration. Structural changes associated with prepared dentin may influence the bonding of restorative materials and most of the studies have established that CMCR produces a roughened surface with altered hardness. ^4^^,^^5^

Glass ionomer cement is still the preferred restorative material for restoring primary teeth, but there are no studies evaluating the bond between the glass ionomer cement with the residual dentin following caries removal using Carie Care ^TM^. The present study was thus planned to evaluate the bond between the resin-modified glass ionomer cement and residual dentin following excavation of carious dentin using Carie Care ^TM^ and conventional caries removal in primary teeth.

## Methods

### Study setting

The study was conducted in the Department of Paediatric and Preventive Dentistry and the Department of Oral Pathology, Manipal College of Dental Sciences, and the Department of Dental Materials at Yenepoya Dental College.

### Study design

The present study was an experimental in vitro study, designed according to the modified Consolidated Standards of Reporting Trials (CONSORT).

### Ethical considerations

All procedures were performed in conformity with the ethical standards of the institutional Ethics committee, Manipal College of Dental Sciences, Mangalore. Ethical clearance was obtained from the Institutional Ethics Committee (ref: 19085 dated 10 ^th^ October 2019) before the study. All the collected teeth were grouped and could not be traced back to any person/child.

### Sample size calculation

The sample size was calculated to be 52 (n=26 in each group) at 90% power, 5% alpha error, and a clinically significant difference of 1 unit.

### Eligibility criteria

52 freshly extracted human primary first and second molars with class I or class II cavitated dentinal lesions with sufficient opening for hand instrumentation were selected for this study. The teeth were selected based on the inclusion and exclusion criteria and were indicated for extraction from patients attending the clinics for dental treatment at the department of Pediatric and Preventive Dentistry, Manipal College of Dental Sciences, Mangalore.

Exclusion criteria included teeth with caries involving the pulp, crack, or defect on the enamel surface.

All the selected teeth were cleaned thoroughly with hand scalers and fluoride-free pumice to remove the extrinsic deposits and blood. The teeth were then stored in 0.1% thymol solution. ^3^^,^^4^

### Outcomes

*Primary outcome:*

1. The bonded interface between resin-modified glass ionomer cement and the treated tooth surface was subjected to modified short-beam shear (MSBS) testing. The micro shear bond strength was calculated using the formula: *Micro shear bond strength (MPa)=Shear Force(N)/Cross-sectional area (mm ^2^).*
2. Microleakage analyses were made by observing the penetration of the dye into the tooth surface through the interface.

*Secondary outcome*

1. The extent of dye penetration into the enamel and/or dentin was noted and recorded.

### Grouping and randomization

**Figure 1.**
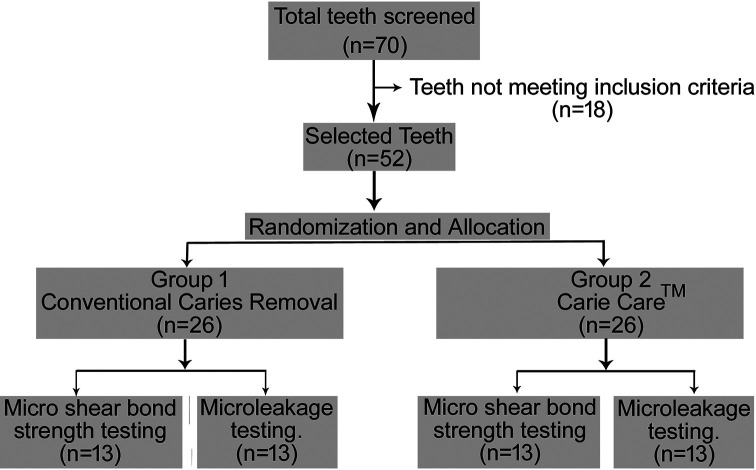
CONSORT diagram.

Based on the inclusion and exclusion criteria, a total of 52 teeth were selected out of the collected 70 teeth ( Figure 1). They were randomly divided into two groups (n=26 teeth in each group) by chit method as follows:

1. Group I – Control group: Conventional caries removal.
2. Group II – Test group: Caries removal using Carie Care ^TM^

Each group was further randomly divided into two subsets (n=13 in each group) using the same randomization technique as follows:

1. Group I and II A – For micro shear bond strength testing.
2. Group I and II B – For microleakage testing.

### Blinding

The random allocation sequence and enrolment of samples were done by a person not involved in the study. The person who tested and evaluated both micro shear bond strength and microleakage and the statistician who carried out the analysis were blinded to the allocation of the samples. However, the operator who had carried out the restorative procedures was not blinded to the allocation.

### Procedure

**Caries removal**

A. **Caries removal by the conventional method**
  Caries was removed by a single operator using a slow-speed contra angled handpiece with large round diamond bur (NMD **Nexus Medodent** Dental Contra Angle Low Speed Handpiece (Latch Type) and 001/018 round bur) under cooling until all the infected dentin was removed. The completeness of the caries removal was checked by running a sharp explorer tip on the floor of the cavity. It should neither give any tug-back sensation nor should stick to the dentin. The caries removal was continued until the criteria were satisfied. ^6^
B. **Caries removal using Carie Care** ^**TM**^
  The gel was placed on the cavitated lesions via the syringe in which it is dispensed. It was left untouched to allow it to work for 60 seconds. When the gel turned cloudy, the softened dentin and the gel were removed using a spoon-shaped hand excavator without applying pressure. The process was repeated until the gel no longer turned cloudy. The completeness of the caries removal was assessed by using the same criteria as for the first group. ^3^

**Restoration of the teeth**

A dentin conditioner (10% polyacrylic acid) was placed on the exposed dentin surface using a micro brush. A PVC tube (internal diameter of about 0.9 mm and 2 mm height) was placed on the dentin conditioner and cured for 10 seconds. RMGIC (GC Gold Label Light Cured Universal Restorative Material, GC Corporation, Tokyo, Japan) was packed compactly inside the tube using a plastic filling instrument, avoiding any voids. It was then cured for 20 seconds using a visible light curing device (Elipar 2500, 3M ESPE, Dental Products, St Paul, MN, USA). A radiometer (Demetron 100, Demetron Research Corp, USA) was used to verify the light intensity of the halogen light-curing device (minimum threshold = 600 mW/cm ^2^). The completely set specimens were stored in distilled water for 24 hours. ^3^

**Sample preparation for micro shear bond strength testing**

**Figure 2.**
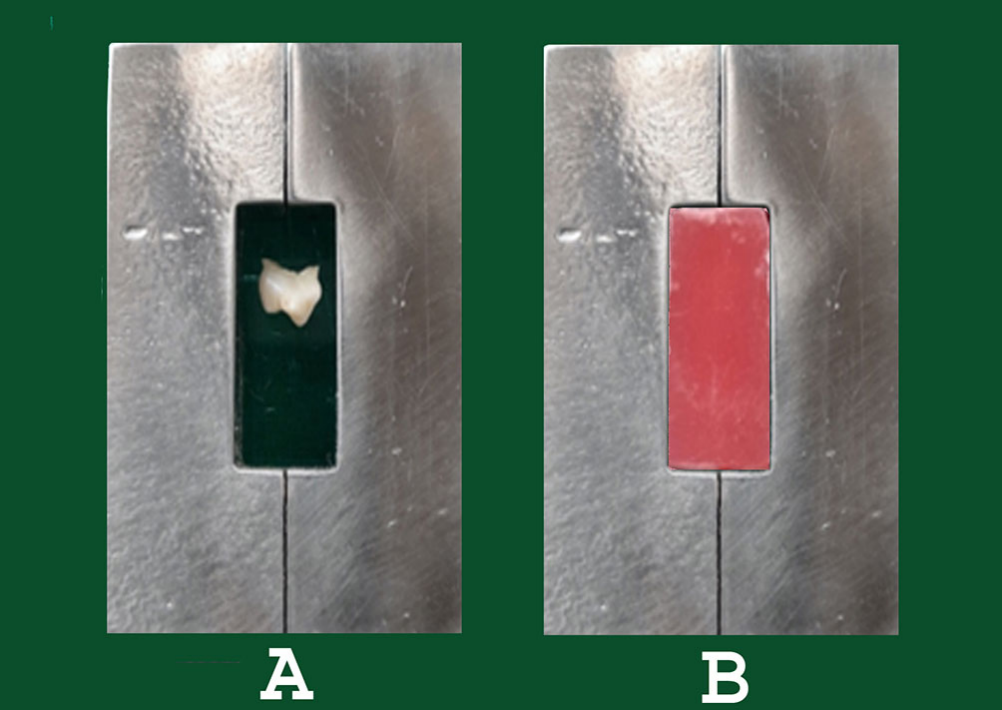
A) Exposed dentinal surface placed touching the glass slab and L-shaped mold placed around the tooth sample for resin block preparation. B) The prepared resin blocks.

Teeth were sectioned at the level of cementoenamel junction to remove the remaining roots with the help of a high-speed handpiece and diamond burs. The specimens were then placed on a glass slide. Two L-shaped molds that when put together created a rectangular space and were used for the fabrication of resin blocks around the specimens. Once the resin was set, the glass slide along with the sticky wax was removed. The resin blocks were then trimmed and polished with 400 and 600-grit silicon carbide (SiC) papers to the desired dimension. The dimensions of the resin block to fit the testing tool jig were approximately 28 mm high, 13 mm wide, and 10 mm thick ( Figure 2A, B) ^3^

**Sample preparation for microleakage**

The teeth were coated with a single layer of air-dry nail varnish (Lakme, India) except at an area approximately 2 mm around the periphery of the restoration. The cervical portion of the teeth was sealed with sticky wax to prevent the seeping of the dye through the cervical aspect. The teeth were placed in 2% methylene blue (Merck KGa A-C.I.52015) for 24 h at room temperature. They were then removed and washed under running water. The teeth were then sectioned in the buccolingual direction using a diamond disc to visualize the penetration of dye at the restoration tooth interface. ^7^

**Microshear bond strength testing**

Testing for micro shear bond strength was done using a universal testing machine (Type: HPBSD, Model no: TSI-BSD-20KN, Serial no: 170710). The samples were fixed onto the jig which in turn was fixed on the mechanical jaw of the micro shear universal testing tool. The bonded interface was then tested using a chisel at a crosshead speed of 1.0 mm/min. ^7^

$$\text{Micro shear bond strength}\;\left(\mathrm{MPa}\right)=\text{Shear Force}\left(N\right)/\text{Cross}-\text{sectional area}\;\left({\mathrm{mm}}^{2}\right)$$

**Microleakage testing**

The degree of dye penetration was scored using a stereomicroscope (ZTX-3E, China) at X20 magnification. The score which was higher was taken as the score for that particular tooth.

**Figure 3.**
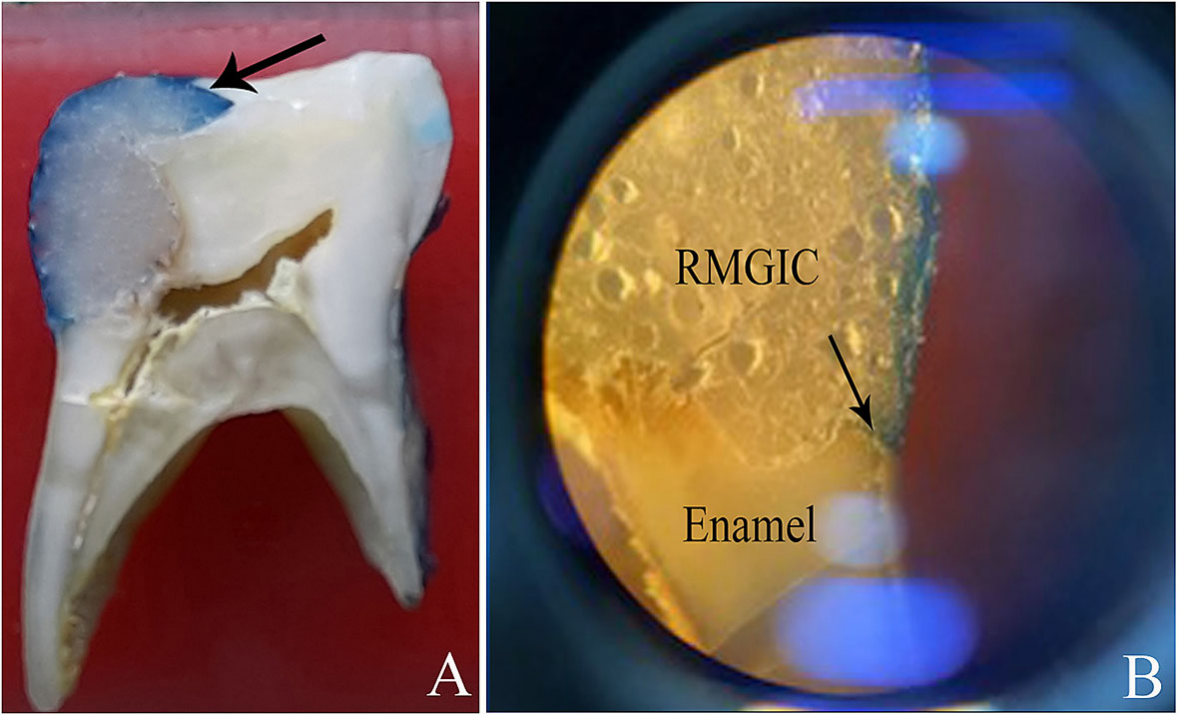
Leakage of the dye at the interface identified by the arrow.

The following scoring criteria were used ^8^ ( Figure 3):

0 – No dye penetration

1 – Dye penetration into enamel only

2 – Dye penetration into the enamel and dentin

3 – Dye penetration into pulp.

### Data management and statistical analysis

After testing both groups for micro shear bond strength and microleakage, the data was entered and analyzed using Statistical Package for Social Science (SPSS), version 20 (SPSS Inc.). An Independent t-test was performed for comparing the mean values of micro shear bond strength and microleakage between the conventional and Carie Care ^TM^ groups. Dye extended into the tooth structure at the restoration interface. The extent of the dye into the enamel/dentin was evaluated using the Pearson chi-square test.

## Results

A total of 52 teeth were divided equally into group I and group II to evaluate micro shear bond strength and microleakage between the resin-modified glass ionomer cement and residual dentin following excavation of carious dentin using Carie Care ^TM^ and by conventional caries removal in primary teeth.

### Outcome 1: Micro-shear bond strength

*Primary outcome*

The highest scores were 10.16 MPa and 14.84 MPa in conventional caries removal and Carie Care ^TM^ groups respectively, and the lowest score in the conventional caries removal group was 4.08 MPa while it was 3.53 MPa in the Carie Care ^TM^ group. The average micro shear bond strength score was 6.03±1.6 MPa in the conventional caries removal group and 8.54±2.92 MPa for the Carie Care ^TM^ group.

Independent t-test revealed a t value of -2.706 and the difference in the mean micro-shear bond strength values between both the groups was statistically significant with a p-value of 0.012 ( Table 1).

**Table 1.** Comparison of mean micro-shear and microleakage of the residual dentin between two groups.

	Control (n=13)	Test (n=13)	t	P-value
	Mean±SD	Mean±SD		
Micro-shear bond strength	6.03±1.6	8.54±2.92	-2.706	0.012
Microleakage	0.77±0.6	1.38±0.51	-2.828	0.009

### Outcome 2: Microleakage

*Primary outcome*

Microleakage was seen in all the samples of the Carie Care ^TM^ group, while 69.20% of the samples in the conventional caries removal group exhibited microleakage. The mean microleakage value of the conventional caries removal group was 0.77±0.6 and that of the Carie Care ^TM^ group was 1.38±0.51.

Independent t-test revealed a t value of -2.828 and the difference in the microleakage values between both the groups was statistically significant with a p *-*value of 0.009 ( Table 1).

*Secondary outcome*

Out of the 9 samples with microleakage in the control group, 8 had leakage into enamel and 1 into enamel and dentin. In group II, 8 samples exhibited microleakage into the enamel, and 5 into enamel and dentin. The microleakage patterns into the enamel and dentin were found to be statistically significant with a p *-*value of .036 ( Table 2).

**Table 2.** Microleakage pattern.

Microleakage extension	Group		
	Group I% (n)	Group II % (n)	Total samples
Nil	30.8% (4)	0 (0)	4
Into the Enamel	61.5 (8)	61.5 (8)	16
Into Enamel and Dentin	7.7 (1)	38.5 (5)	6
**Total**	**100 (13)**	**100 (13)**	**26**
**Pearson chi-square**	**Value**	**df**	**P value (<0.05 significant)**
	6.667	2	**.036**

## Discussion

The chemo-mechanical caries removal (CMCR) method makes use of a chemical that softens the degraded collagen fibers in the infected dentin which is then easily removed by gentle mechanical action by a hand instrument without affecting the healthy tissues. There are two types of chemomechanical caries removal agents, sodium hypochlorite-based and papain enzyme based. Papain is a proteolytic enzyme, derived from the latex of the papaya leaves and fruit with bactericidal, bacteriostatic as well as anti-inflammatory properties similar to the actions of the human pepsin enzyme. It acts as a debriding agent and doesn't impair healthy tissues. Examples of this system are Papacárie ® and Carie Care ^TM^. Carie Care ^TM^ is used in the present study and is relatively new, simple to use and does not require any training or any special equipment for its use, and is much more economical. Other components of Carie Care ^TM^ are chloramine, gelling agents and clove oil, colored gel (blue), sodium chloride, and sodium methylparaben. ^3^ Chloramines help in the healing process and shorten tissue repair time and have the potential to dissolve carious dentin through chlorination of partially degraded collagen. This helps in the disruption of collagen structure, dissolves hydrogen bonds, and helps in tissue removal. Clove oil has an analgesic and antiseptic action. Sodium methylparaben is used as a preservative. ^9^^–^^12^

Anwar *et al.* ^5^ in their study found that the microhardness (KHN) of the residual dentin following Carie Care ^TM^ application was reduced compared to that following caries removal using burs.

There is no existing literature evaluating the bond between resin-modified glass ionomer cement and residual dentin treated with a papain-based CMCR agent, Carie Care ^TM^ in primary teeth. The most cited failures of restoration are lack of marginal adaptation and loss of retention. ^13^ RMGIC was chosen in the present study as it is the most preferred material for the restoration of primary teeth. ^14^

The present study was thus initiated to evaluate the bond between the residual dentin and resin modified glass ionomer cement following caries removal with Carie Care ^TM^ and the conventional method.

In vitro tests have many advantages such as simplicity, ease of sampling for microleakage, etc. Shear bond strength testing is considered to be one of the most commonly used methods for testing bond strength, especially for any substrate susceptible to crack propagation during sample preparation like glass ionomer cements. ^15^ Micro shear bond strength testing was used in the present study as it results in a uniform stress distribution over a small area (<1 mm ^2^) leading to more reliable results. ^16^

The current study demonstrated that the micro shear bond strength between Carie Care ^TM^ treated residual dentin and RMGIC was significantly higher than the conventional caries removal group. This finding was different from that of other studies which were done on permanent teeth. ^3^ Many earlier studies on permanent teeth using an earlier system of CMCR showed that the chemomechanical method did not influence the bond strength. ^3^^,^^17^

Caries removal using low-speed rotary instruments produced a smooth and uniform smear layer over the dentin surface while the dentin exhibited intertubular microporosity with minimal or no smear layer, exposing the dentinal tubules following the use of Carie Care ^TM^ with fibrous structure inside the tubules in primary molars. The presence of open dentinal tubules in chemo-mechanical caries removal is attributed to the initial high pH of the gel due to the presence of chloramine. ^18^

Bonding could also vary depending on the orientation and density of the dentinal tubules. The infiltration of the restorative material is higher in the deeper regions of dentin because of the wider dentinal tubules and perpendicular orientation of the tubules to the pulp wall. Likewise, the bonding in dentin is better in the proximal walls as compared to the occlusal wall. ^19^ These factors could explain the differing results of other studies compared to this study.

The rationale of testing microleakage is that it can be considered as a proxy for the penetration of bacteria and fluids along the restoration-tooth interface intraorally which may result in hypersensitivity, secondary caries, pulpitis, etc. There are several methods of evaluating microleakage and one of them is the dye penetration method using dyes like methylene blue, rhodamine, or erythrosine. It is simple, inexpensive, doesn't require the use of complex chemicals and testing equipment, and also allows the investigator to view the longitudinal sections but in a two-dimensional view. ^20^

In the current study, the mean microleakage values in the Carie Care ^TM^ group were higher compared to the conventional caries removal group and extended into the dentin, which is similar to other studies. ^21^^,^^22^

Khattab & Omar *et al.* ^21^ concluded that glass ionomer exhibited more microleakage and lower micro shear bond strength than composite resin restoration after the use of Papacarié gel in primary teeth.

Carie Care ^TM^ was found to be easy to handle, easy and efficient for caries removal, and provides good bond strength with resin-modified glass ionomer cement. The drawback of Carie Care ^TM^ is that it provides no improvement in microleakage which is one of the main drawbacks of CMCR agents.

The following conclusions can be drawn from the present study:

1. The mean micro shear bond strength following carious removal using Carie Care ^TM^ was found to be better compared to the control group in which caries were removed by the conventional method.
2. Carie Care ^TM^ exhibited more microleakage compared to that of the control group and extended into the enamel and dentin.

### Limitations of the study

1. In the present study all primary teeth with class I or class II caries were selected. The depth of the carious lesion, the lesion activity, the shape and location of the lesions, and the consistency of the dentin could not be standardized, which could have influenced the results.
2. Extracted teeth may respond very erratically to the caries excavation compared to vital teeth, because of the outward flow of dentinal fluid in the tubules in vital teeth. The future scope of the present study may be to observe structural changes in the dentin of primary teeth following caries removal using Carie Care ^TM^ to obtain more insight.

**Key points**

• Carie Care ^TM^ may be a better choice over other chemomechanical caries removal systems as they do not affect the bond strength of restorative material.
• Microleakage is associated with Carie Care ^TM^ similar to other chemomechanical caries removal systems.

## Data Availability

figshare: Microshear bond strength Raw Data, https://doi.org/10.6084/m9.figshare.22213999.v1. ^23^ figshare: Raw Data-Microleakage, https://doi.org/10.6084/m9.figshare.22214011.v1. ^24^ figshare: Figure 1. https://doi.org/10.6084/m9.figshare.22140266.v1. ^25^ figshare: Figure 2. https://doi.org/10.6084/m9.figshare.22140293.v2. ^26^ figshare: Figure 3. https://doi.org/10.6084/m9.figshare.22140296.v3. ^27^ Data are available under the terms of the Creative Commons Attribution 4.0 International license (CC-BY 4.0). figshare: Modified CONSORT checklist for reporting in vitro studies of dental materials for ‘A comparative evaluation of micro shear bond strength and microleakage between the resin-modified glass ionomer cement and residual dentin following excavation of carious dentin using Carie CareTM and conventional caries removal in primary teeth: an in vitro study’. https://doi.org/10.6084/m9.figshare.22140305.v1.
