# Supplementary material for: A comparative evaluation of micro shear bond strength and microleakage between the resin-modified glass ionomer cement and residual dentin following excavation of carious dentin using Carie CareTM and conventional caries removal in primary teeth: an in vitro study
Source: F1000Res. 2023 Mar 24;12:332. [Version 1] doi: 10.12688/f1000research.131919.1 (PMC10206442; doi:10.12688/f1000research.131919.1)
Supplement: Peer review history

# Peer review: Reviewer response for version 1

**DOI:** 10.5256/f1000research.144808.r169584

**Published:** 2023-05-23

**Authors:** Kushal Vasanth Shetty

**Affiliations:** 1 Department of Pedodontia, A.J. Institute of Dental Sciences, Mangalore, Karnataka, India

The topic of the study has been put forth well in the article. Newer methods of caries excavation have been practiced to avoid excess unnecessary removal of the sound dentin. The study has been done meticulously and written excellently. At the same time, the author has addressed the needed concern- will the residual dentin bond adequately with the restorative material and for the same reasons based on my level of expertise, I suggest that the article meets the criteria to be indexed.

 Following are my comments after reviewing the article.

**Abstract**

 The abstract is well structured and is summarized adequately. It is precise and does not need any revision

**Introduction**

 The authors have clearly mentioned the purpose of the study with clear background. It is brief and gives a clear introduction for further reading.  Most of the studies are performed on permanent teeth, Since glass ionomer cement is the most frequently used restorative material in primary teeth, choice of the material is the need of the time especially when used following a newer chemomechanical caries removal system in a primary teeth.

**Materials and Methods**

 The method described is self explanatory and very easy to reproduce which should be the hallmark of any study. The sectioned mentioned in the materials and method makes it clear for any reader to understand. Some of the mentions in the article such as the anonymity aspect of the extracted teeth, primary and secondary outcomes, randomization and blinding are the positive observations,

 Authors could have mentioned the period for which the teeth were stored before caries removal.

**Results**

 The results are clearly mentioned and explained with tables.

**Discussion**

 The authors have briefly mentioned the types of CMCR with clear explanation of the current study material and its components. Reasons for choosing the restoration material in a primary material is well explained.  Authors have also included the discussion on the rationale for choosing the different tests used.

 The outcome of the study is discussed with studies having same and contradictory outcomes.

 By mentioning the limitations of the study, authors have hinted at the scope for the improvement of the study. Key points suggests to the researcher that future study is needed in CMCR to improve its properties and minimizing microleakage.

Is the work clearly and accurately presented and does it cite the current literature?

Yes

If applicable, is the statistical analysis and its interpretation appropriate?

Yes

Are all the source data underlying the results available to ensure full reproducibility?

Yes

Is the study design appropriate and is the work technically sound?

Yes

Are the conclusions drawn adequately supported by the results?

Yes

Are sufficient details of methods and analysis provided to allow replication by others?

Yes

Reviewer Expertise:

Dental research

I confirm that I have read this submission and believe that I have an appropriate level of expertise to confirm that it is of an acceptable scientific standard.

# Peer review: Reviewer response for version 1

**DOI:** 10.5256/f1000research.144808.r168089

**Published:** 2023-05-10

**Authors:** Parajeeta Dikshit

**Affiliations:** 1 Department of Pedodontics, Kantipur Dental College, Kathmandu, Nepal

The paper is written excellently, and is well organized with detailed descriptions in all sections. The article addresses an important topic that has been minimally researched and will be an important milestone for research in dentistry. Based on my level of expertise the article meets the criteria to be published with minimal revisions.

 Following are my comments and suggestions after reviewing the allotted article.

**Abstract**

 The abstract has summarized the research adequately and does not have scope for any more revision. The methods section in abstract begins with a number which can be rectified by mentioning the number in words. The abstract could have a clearer conclusion.

**Introduction**

 The introduction is brief encompassing the highlights of what to expect from the present study. It has mentioned about the materials and techniques used adequately. The authors are suggested to mention about the history of chemomechanical caries removal technique with special mention about the type of glass ionomer cements used in primary teeth and why Resin modified Glass ionomer cements are preferred over the others.

**Materials and Methods**

 This section has been meticulously written describing each step in detail which will be an asset for future research. There are with only few minor suggestions as mentioned below:

- The authors are suggested to mention the age group of children whose teeth were included in the study
- The time period for storage of teeth after extraction and before preparation is suggested to be mentioned.
- There could have also been a mention about the criteria for teeth selection based on range of depth of the caries lesion with classification of it.

 
**Results**

 The result can be published as it is as the data has been analyzed well and described with necessary tables.

**Discussion**

 The article has sufficiently discussed all aspects of the research with the comparison of materials used as well as the technique. Cross references with similar as well as contrasting research has been appropriately described by the authors. This study is an initiative to evaluate the bond between residual dentin and resin modified glass ionomer cement which will be a landmark for future research in this category. The limitations have been mentioned by authors for future studies.

Is the work clearly and accurately presented and does it cite the current literature?

Yes

If applicable, is the statistical analysis and its interpretation appropriate?

I cannot comment. A qualified statistician is required.

Are all the source data underlying the results available to ensure full reproducibility?

Yes

Is the study design appropriate and is the work technically sound?

Yes

Are the conclusions drawn adequately supported by the results?

Yes

Are sufficient details of methods and analysis provided to allow replication by others?

Yes

Reviewer Expertise:

Pediatric Dentistry, Child Psychology, Dental Caries, Dental Materials, Gingival diseases, Behaviour Management, Orthodontics in Children, Pediatric Endodontics, Trauma Management of Anterior teeth.

I confirm that I have read this submission and believe that I have an appropriate level of expertise to confirm that it is of an acceptable scientific standard.
